# RNAi-Mediated Knockdown Showing Impaired Cell Survival in *Drosophila* Wing Imaginal Disc

**DOI:** 10.4137/grsb.s2100

**Published:** 2009-02-19

**Authors:** Makoto Umemori, Okiko Habara, Tatsunori Iwata, Kousuke Maeda, Kana Nishinoue, Atsushi Okabe, Masahiko Takemura, Kuniaki Takahashi, Kaoru Saigo, Ryu Ueda, Takashi Adachi-Yamada

**Affiliations:** 1 Department of Biology, Graduate School of Science, Kobe University, Kobe, Japan; 2 Japan Science and Technology Agency, Japan; 3 Department of Sciences for Natural Environment, Graduate School of Cultural Studies and Human Science, Kobe University, Kobe, Japan; 4 Department of Sciences for Natural Environment, Faculty of Human Development, Kobe University, Kobe, Japan; 5 Genetic Strains Research Center, National Institute of Genetics, Mishima, Japan; 6 Department of Biophysics and Biochemistry, Graduate School of Science, The University of Tokyo, Tokyo, Japan

**Keywords:** Drosophila, RNAi apoptosis, JNK caspase-3

## Abstract

The genetically amenable organism *Drosophila melanogaster* has been estimated to have 14,076 protein coding genes in the genome, according to the flybase release note R5.13 (http://flybase.bio.indiana.edu/static_pages/docs/release_notes.html). Recent application of RNA interference (RNAi) to the study of developmental biology in *Drosophila* has enabled us to carry out a systematic investigation of genes affecting various specific phenotypes. In order to search for genes supporting cell survival, we conducted an immunohistochemical examination in which the RNAi of 2,497 genes was independently induced within the dorsal compartment of the wing imaginal disc. Under these conditions, the activities of a stress-activated protein kinase JNK (c-Jun N-terminal kinase) and apoptosis-executing factor Caspase-3 were monitored. Approximately half of the genes displayed a strong JNK or Caspase-3 activation when their RNAi was induced. Most of the JNK activation accompanied Caspase-3 activation, while the opposite did not hold true. Interestingly, the area activating Caspase-3 was more broadly seen than that activating JNK, suggesting that JNK is crucial for induction of non-autonomous apoptosis in many cases. Furthermore, the RNAi of essential factors commonly regulating transcription and translation showed a severe and cell-autonomous apoptosis but also elicited another apoptosis at an adjacent area in a non-autonomous way. We also found that the frequency of apoptosis varies depending on the tissues.

## Introduction

In recent years, mechanisms controlling apoptosis have been extensively studied, and various factors are known to be involved in the intrinsic and extrinsic apoptotic pathways.^1^–^4^ Although these pathways play a pivotal role in the execution of most cases of apoptosis, the apoptosis shown in developing animal tissues is also affected by various growth and differentiation signals to promote or repair organ development.^5^ In general, inhibition of apoptosis accompanies growth induction, whereas reduction of growth conversely leads to apoptosis. However, we can often find exceptions showing an opposite relationship, such as overgrowth-induced apoptosis^6^–^8^ and apoptosis-induced overgrowth,^9^–^11^ indicating that we do not fully understand these cell survival controls between apoptosis and growth. In order to systematically investigate the apoptosis phenotype caused by reducing each gene function in the developing animal tissues, we employed a genetically amenable fruit fly *Drosophila melanogaster*, in which each gene can be knocked down by RNAi,^12^–^14^ to observe the effect on apoptosis induction. RNAi provides an easy and powerful technique for reducing the quantity of mRNA derived from endogenous specific genes, and it has recently been applied in many studies to investigate various gene functions.^15^

In this study, we screened 2,497 protein-coding genes of *Drosophila* to determine whether they were required for prevention of apoptosis in the wing imaginal disc and found that 47% of them showed an apoptosis induction when their functions were knocked down by RNAi in the developing wing disc. Most of the cases (82%) with detectable Caspase activation were associated with JNK activation, which was unexpectedly high because JNK has not been observed as essential for all apoptosis. Alternatively, JNK is known to be involved in inducing non-autonomous apoptosis,^16^,^17^ which occurs in cells distant from the cells associated with the primary cause of apoptosis. Interestingly, a major part of the JNK and Caspase-3 activation found in this study occurred in a non-autonomous manner, suggesting that the non-autonomous pathway is a common way to induce apoptosis. Loss of membrane proteins frequently caused JNK activation, which had also been expected because cell-cell communication is presumed to be important for many developmental processes, including apoptosis in multicellular organisms. These results, as well as the database showing the immunofluorescent data, provide an archival source for survey of genes and for fine analysis of each gene in apoptosis regulation using the *Drosophila* imaginal discs.

## Results

### Rationale for RNAi-mediated screening for genes regulating apoptosis

We induced RNAi in the dorsal compartment of the wing disc and monitored the activities of Caspase-3 and JNK. Caspase-3 plays a central role in most apoptosis, while JNK leads to a subgroup of stress-induced apoptosis.^18^ In the *Drosophila* wing disc, JNK activation is usually linked to the activation of Caspase-3.^16^ Puc is a protein phosphatase specifically inactivating JNK, and its transcription occurs in response to the JNK signal, thereby making a negative-regulatory circuit.^19^ Thus, the expression of *puc* reflects the JNK activity and can be used for monitoring it.

**Figure 1:**
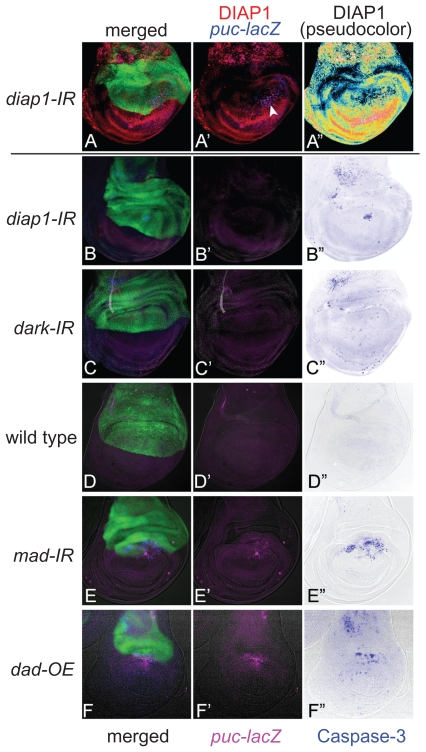
Similarity of RNAi and conventional gene manipulation in their apoptotic phenotypes. (A–A″) RNAi of *diap1* by expressing its inverted repeat (IR) sequence in the dorsal compartment displayed a reduction of DIAP1 protein levels specifically in the dorsal compartment. Expression of a dorsal compartment marker *apterous* (green), DIAP1 (red), and JNK indicator *puc-lacZ* (blue) are shown. A″ shows a pseudocolor image representing DIAP1 protein levels. Arrowhead in A′ indicates a cluster of high level staining of DIAP1 and *puc-lacZ*, which is caused by the presence of massive apoptotic bodies. (B–F) Expression of *apterous* (green), *puc-lacZ* (magenta), and activated Caspase-3 (blue) are shown. (B–B″) RNAi of *diap1* in the dorsal compartment led to an activation of Caspase-3 at the specific position in the dorsal compartment. When the contrast of B′ image is elevated, *puc-lacZ* expression can be observed at around the apoptotic bodies as in A′ (not shown). (C–C″) RNAi of *dark* in the dorsal compartment as a negative control experiment. No activation of JNK and faint activation of Caspase-3 were observed. (D–D″) Wild type. JNK and Caspase-3 are not activated in wild type. (E–E″) RNAi of *mad* in the dorsal compartment led to a non-autonomous activation of JNK and Caspase-3 in the medial region of the DV boundary. (F–F″) Overexpression (OE) of *dad* in the dorsal compartment, which results in a reduction of Dpp signaling, induces a shrinkage of the dorsal compartment as well as a non-autonomous activation of JNK and Caspase-3 in the medial region of the DV boundary.

Before expanding the RNAi analyses to the entire genome, we checked whether the mutant phenotypes caused by previously known apoptosis-regulating genes, such as *diap1*^20^ and *dark,*^21^–^23^ were reproduced by their RNAi. When *diap1* (*Drosophila* Inhibitor of Apoptosis Protein 1) was knocked down within the dorsal compartment of the imaginal disc, a local but prominent activation of Caspase-3 was detected (Fig. 1B). The position-specificity may be dependent on the difference in sensitivity in the induction of apoptosis, as described later. In contrast, when *dark* (*Drosophila* Apaf-1-Related Killer) was knocked down, no apoptosis induction was observed (Fig. 1C). Furthermore, the use of this collection of RNAi strains has already been validated, since they were screened for apoptosis phenotype in the compound eye.^24^

For the non-autonomously induced apoptosis during restoration of morphogenesis, for example, we tested whether the apoptosis shown in several RNAi samples really reflected the non-autonomous apoptosis by conventional gene manipulation in previously studies.^16^ We manipulated signaling factors for a diffusible extracellular ligand Dpp, a homolog of mammalian BMP (Bone Morphogenetic Protein)–2/4. Mad (Mothers against Dpp),^25^ a *Drosophila* homolog of mammalian r-Smad, transmits the intracellular signal caused by Dpp. As shown in Figure 1E, the RNAi of *mad* within the dorsal compartment activated JNK and Caspase-3 in both dorsal and ventral compartments of the central wing disc region, which is a typical example of non-cell-autonomous induction of apoptosis. These features are also quite similar to those seen in the case of overexpression of Dad (Daughters against Dpp), a homolog of anti-Smad, (Fig. 1F) that can repress Dpp signaling.^26^ Therefore, RNAi can mimic the conventional gene manipulation in induction of apoptosis, at least in some representative examples.

We describe how to choose the genes for RNAi in the section on Experimental Procedures. During observation of our RNAi results, we focused on six areas of the wing disc (Notum [N], Dorsal wing Blade [DB], DorsoCentral spot [DC], Dorsal wing Margin [DM], Ventral wing Margin [VM], and Ventral wing Blade [VB]), and classified the strength of JNK and Caspase-3 activities in each area into three grades (+ −, +, and +). The database was constructed by using FileMaker Pro 7 (FileMaker, Inc.) and contains each immunofluorescence image with the above classification of JNK/Caspase-3 activities in each gene page.

**Figure 2:**
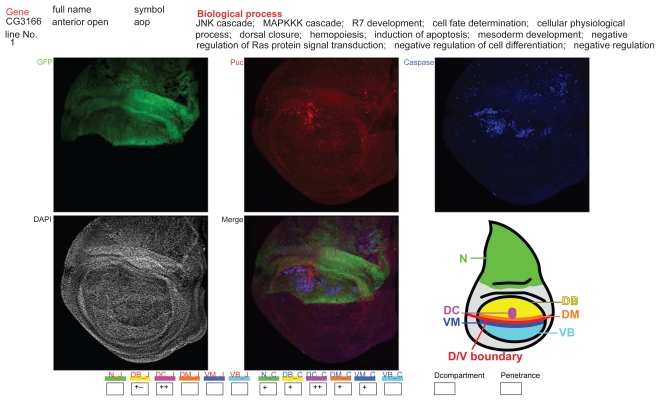
Web page showing the database for immunofluorescent images of the imaginal discs. The page for *anterior open* (*aop*) is shown here as an example. The gene’s full name, CG number, gene symbol, strain line number, and biological process are described at the top according to the Flybase (http://flybase.bio.indiana.edu/). The top three photographs are images for each color channel (green [*apterous*], red [JNK], and blue [Caspase-3]). The center-bottom photograph shows an image merged with the three colors. In the left-bottom photographs, the DAPI staining is displayed to recognize the position and shape of the imaginal discs in the other photos. In some photographs, three kinds of imaginal discs (leg, haltere and wing) are shown in a single frame. In the right-bottom corner of the figure, abbreviations for the position in the wing imaginal discs are indicated using a cartoon. The colors used in this cartoon are not related to those in the immunofluorescent images. Small boxes at the bottom box row indicate the levels of JNK and Caspase-3 activation by symbols as follows: +−, +, ++. In this example, strong activation of JNK and Caspase-3 can be seen only around the DC spot area in the wing blade region. The abbreviations are explained in the fourth paragraph of the Results section.

First, we noticed that a narrow area in the dorsocentral (DC) wing region showed JNK and Caspase-3 activation too sensitively (e.g. Fig. 2), which was not always correlated with RNAi. Consequently, the results that simply reflected this feature were excluded from all of the analyses.

**Figure 3:**
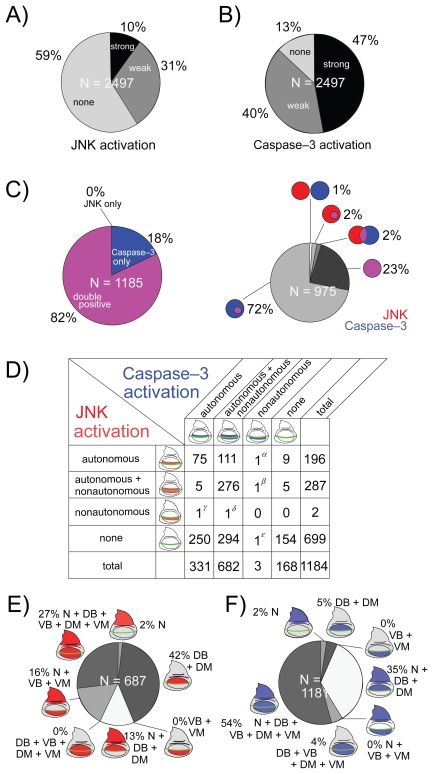
Categorization of RNAi-induced JNK and Caspase-3 activation. **A**) Frequency of genes in which RNAi induces JNK activation. Cases with activation only at the dorsocentral spot (e.g. *aop* shown in Fig. 2) are excluded. **B**) Frequency of genes in which RNAi induces Caspase-3 activation. Again, cases with activation only at the dorsocentral spot are excluded. **C**) Relationship between JNK and Caspase-3. Cases showing strong activation of at least either JNK or Caspase-3 are analyzed. Left: Overlap of JNK and Caspase-3 activation. In most cases, both are simultaneously activated. Caspase-3 are sometimes activated solely (e.g. Fig. 1B), whereas JNK activation without Caspase-3 activation is rare. Right: Pattern of relative position of JNK-activating area (red) and Caspase-3-activating area (blue). Overlapping areas are shown in magenta. **D**) Relation of cell autonomy in JNK activation and that in Caspase-3 activation around the DV boundary in the wing blade. Examples showing rare patterns in the table are *RpL17* (α), *shotgun* (β), *CG14122* (γ), *CG14072* (δ), and *E(spl)m5* (ɛ). **E**) Frequencies of each JNK activation pattern with regard to wing disc subdomains. **F**) Frequencies of each Caspase-3 activation pattern with regard to wing disc subdomains.

When a survey of all of the RNAi experiments was carried out, both weak and strong activation of JNK and Caspase-3 (shown by + and ++ signs in the database) was observed in 41% and 87% of the cases, respectively (Fig. 3A). These proportions seemed much higher than expected because the imaginal disc cells may not actually express such a large number of genes, and they are thought to express several thousand genes.^27^–^29^ This large number of strains with weak JNK and/or Caspase-3 activation may reflect false-positive results due to an off-target effect (OTE) or other non-specific effects of dsRNA expression. A part of these IR lines was known to have possible OTs and the frequency of such suspected OTE lines was calculated to be 47% as a maximal estimation. Accordingly, we did not give further consideration to this class of strains and hereafter focused on the results showing high levels of JNK and Caspase-3 activation (shown only by the ++ sign in the database), which amounted to 10% and 47% of cases, respectively (Fig. 3A). The immunofluorescence data can be accessed on the website: http://www.shigen.nig.ac.jp/fly/nigfly/index.jsp (see Experimental Procedures).

### Screening summary

Among these strong cases of JNK and/or Caspase-3 activation, more than 80% showed a coupling of JNK and Caspase-3 activation to various extents (Fig. 3C). Conversely, there were only two cases in which JNK activation did not accompany Caspase-3 activation (calculated as 0.17%). Therefore, these findings are consistent with the previous observation that the JNK activation precedes Caspase-3 activation and is strongly linked to apoptosis in the *Drosophila* wing.^17^ Furthermore, we carefully assessed the non-cell autonomous effect of RNAi by examining the phenotypes in the vicinity of the DV boundary. Most of the RNAi-induced JNK/Caspase-3 activation showed a striking non-cell autonomy (Fig. 3D), which is similar to the previously known feature in non-cell-autonomous activation of JNK by altered Dpp signaling.^17^ This suggests that the non-cell-autonomous induction of apoptosis is one of the common patterns in cell death induction.

It has previously been shown that LRR (Leucine Rich Repeat) family cell adhesion proteins contribute to unique cell affinity. Furthermore, alteration of LRR protein functions causes JNK activation followed by Caspase-3 activation.^30^,^31^ Therefore, transmembrane and secreted proteins are good candidates for mediators of non-cell-autonomous apoptosis. We thus tested the apoptosis-inducing activity by RNAi of putative secreted proteins selected based on the presence of N-terminal signal sequences. Consistently, the RNAi of such genes caused a higher frequency of strong JNK activation (74 out of 296 cases, 25%) when compared with the frequency of strong JNK activation in all of the RNAi cases (248 out of 2,497 cases, 10%). Moreover, the RNAi of various transcription factors frequently showed non-autonomous apoptosis, suggesting that they are highly involved in regulation of morphogenesis and that their aberration likely induces non-autonomous apoptosis. These results suggest that a relatively large number of secreted proteins and transcription factors are involved in the prevention of non-cell-autonomous apoptosis. However, functional redundancy may have prevented identification of such molecules.

**Figure 4:**
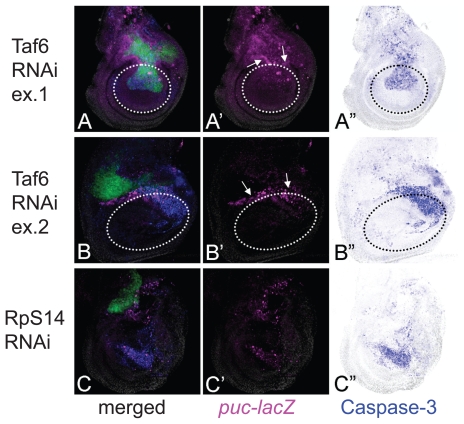
Examples showing non-autonomous activation of JNK and Caspase-3. (A–A″) An example of Taf6 RNAi. A non-autonomously induced JNK activation around the DV boundary in the hinge region is indicated by arrows. The approximate position of the boundary between the wing blade and hinge regions is indicated by the circle with the broken line. (B–B″) Another example of Taf6 RNAi, which shows a more severe autonomous apoptosis in the dorsal cells in the wing blade region. (C–C″) RNAi of RpS14. Similar to B–B″, a severe autonomous apoptosis can be observed. *puc-lacZ* expression (magenta). Caspase-3 activation (blue).

We also noted that there was a significant tendency for various examples of RNAi-mediated apoptosis to be preferentially found in the wing blade region but not outside of this region. For example, the RNAi of *taf6* (TBP-Associated Factor 6) leads to an autonomous activation of Caspase-3 at high levels in the wing blade region but at lower levels in the wing hinge region (Fig. 4). Similar traits have also been reported in the case of the apoptosis induced by a reduced-Dpp signal.^17^ Furthermore, when we surveyed all of the results, the activation of JNK/Caspase-3 in the wing blade was found to be much more frequent than those in the notum (Figs. 3E and F). Consequently, localization of apoptosis in the wing blade region may not be a feature specific to the alteration of a particular cell signal (such as Dpp) but instead a general feature found in all cases of apoptosis in the wing disc. This suggests that the number of stimuli to activate JNK or apoptosis varies depending on the tissue, or that the sensitivities to alterations of gene expression in induction of apoptosis are quite different between tissues.

### Non-autonomously induced apoptosis

As we reported previously, aberrations of some morphogenetic signaling induce JNK activation followed by Caspase-3 activation at the boundary between cell populations with different levels of signaling intensities. This non-autonomous apoptosis is thought to be important for restoration of abnormally developing tissues.^17^ Various examples of apoptosis are probably induced in a similar non-autonomous way. To determine to what extent this applies to non-autonomous apoptosis, we surveyed the relationship in cell autonomy between JNK and Caspase-3 activation by focusing on the DV boundary at which the two cell populations come in contact (Fig. 3D). Around this position, there is an apparent tendency for JNK and Caspase-3 activation to occur simultaneously, which was observed in 471 cases, as shown in the 9 upper-left boxes in Fig. 3D’s grid. As stated above, the most frequent pattern is that both JNK and Caspase-3 are both autonomously and non-autonomously activated. However, 112 out of 196 cases with autonomous JNK activation (57%) displayed a non-autonomous Caspase-3 activation (upper-most row in Fig. 3D grid). In contrast, except for 6 cases, autonomous Caspase-3 activation (331 cases) did not show non-autonomous JNK activation (leftmost column in Fig. 3D grid). Accordingly, these data strongly suggest that JNK activation is also crucial for priming non-autonomous apoptosis, whereas Caspase-3 is not.

Furthermore, when observed throughout the wing disc, the activation patterns of JNK and Caspase-3 are different (Figs. 3E and F). JNK activation seems to be found unevenly in the dorsal region (2 + 42 + 13 = 57%), whereas the Caspase-3 activation only within the dorsal region is less (2 + 5 + 35 = 42%). On the other hand, JNK with non-autonomous activation is minor (0 + 0 + 16 + 27 = 43%), whereas non-autonomous Caspase-3 is major (0 + 0 + 4 + 54 = 58%).

We were interested in the fact that some RNAi examples resulted in a non-autonomous apoptosis similar to that seen previously.^17^ The RNAi of basal transcription factor Taf6 showed an autonomous activation of Caspase-3 in the blade region and a non-autonomous activation of JNK in the hinge region (Figs. 4A, B), the latter of which was unexpected because Taf6 is known to be necessary for the function of TBP (TATA-Binding Protein), suggesting its ubiquitous requirement for most of the transcription by RNA polymerase II. Accordingly, the RNAi of *taf6* is expected to induce a severe autonomous apoptosis in all of the tissues, as is the case for the RNAi of ribosomal protein genes (e.g. RpS14, Fig. 4C). Thus, the non-autonomy in JNK activation in the hinge region in *taf6* RNAi suggests a morphogenetic function rather than its common transcriptional function and/or a difference in the sensitivity of decreased transcription leading to apoptosis between the tissues. The difference in responses between the blade and hinge regions was previously described in the apoptosis associated with homeotic transformation by overexpression of *spineless.*^30^

**Figure 5:**
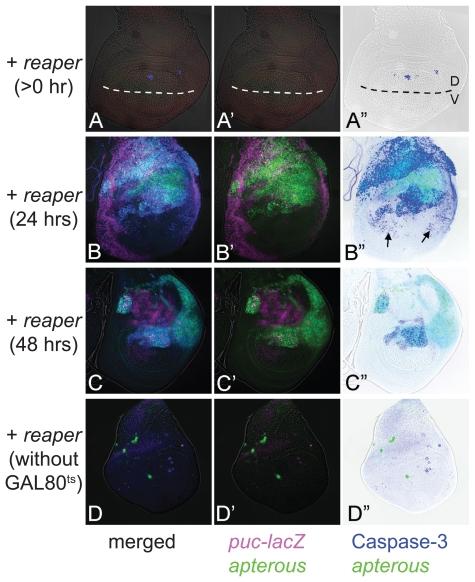
Induction of autonomous apoptosis in the wide area of the wing disc leads to a non-autonomous apoptosis in an adjacent position. (A–A″) Just after induction of *rpr* expression. Active Caspase-3 is observed in the dorsal compartment. *UAS-GFP* expression in the dorsal compartment is not visualized yet at this point. Broken lines indicate the position of the DV boundary. (B–B″) 24 hrs after induction of *rpr* expression. A non-autonomously induced Caspase-3 activation is indicated by the arrows. (C–C″) 48 hrs after induction of *rpr* expression. Non-autonomous Caspase-3 activation is no longer induced. (D–D″) Induction of *rpr* expression without GAL80^ts^. Most of the area in the dorsal compartment has already disappeared. *puc-lacZ* expression (magenta). Caspase-3 activation (blue).

Through these RNAi experiments, we discovered numerous cases of non-autonomous apoptosis cases. Among these, there are particular cases in which an autonomous apoptosis must be induced as a primary response (e.g. *RpL17*, *shotgun*) while an additional non-autonomous apoptosis may be further induced as a secondary response, which is probably caused by a juxtaposition of a normal area and a wide apoptotic area, as proposed previously.^32^ Therefore, we tested a model case in which the proapoptotic gene *reaper* (*rpr*) is temporarily induced within the dorsal compartment by combination with a temperature sensitive-GAL80^33^ (Fig. 5). As a result, at around 24 hours after *rpr* expression, non-autonomous apoptosis could be observed in the ventral compartment (Fig. 5B), although no similar non-autonomy was observed earlier or later (Figs. 5A and C). Thus, a wide area of autonomous apoptosis induction can cause a secondary non-autonomous apoptosis. This phenomenon seems to be a repair mechanism for fitting the adjacent tissue size.^32^

## Discussion

We created a database to show the immunofluorescent images for JNK and Caspase-3 activities in each RNAi experiment. In addition to our purpose of surveying genes affecting cell survival, the database may also be useful for searching for genes regulating tissue growth and patterning. For example, the RNAi of *mad* displays an apparent shrinkage of the compartment size without showing severe apoptosis except at the DV boundary (Fig. 1E). This phenotype strongly suggests the involvement of this gene in tissue growth and/or patterning. In contrast, the wing disc in which the dorsal compartment cells overexpress *rpr* showed a wide and severe apoptosis so that most of the dorsal cells disappeared (Fig. 5D). This case represents a typical phenotype, showing that the gene plays a role exclusively for apoptosis. These findings provide insight into the roles of genes for regulating various developmental processes.

As is the case for the above-mentioned possibility of weak activation of JNK and/or Caspase-3, the OTE should also be considered for all phenotypes.^34^ As an effective initial examination, the RNAi phenotype must be ameliorated by addition of the wild type transgene that is targeted by RNAi. Furthermore, two ways to distinguish the real RNAi effect from OTE have been proposed.^35^ One is a test of a dsRNA corresponding to the other part of the same mRNA for displaying the same phenotype. The other is a test of an artificially altered transgene that is not targeted by the dsRNA but that encodes the same amino acid sequence for complete rescue of the phenotype. Of course, classical analysis using the loss-of-function mutant may be another reliable way to judge the involvement of the gene in each phenotype. In either case, further analyses are required to demonstrate each phenotype as a real loss-of-function phenotype of the gene under focus. The database will be updated when we check the phenotype by the above examinations or accumulate the data from other RNAi constructs.

## Experimental Procedures

### Materials

Various fly strains harboring a transcribable inverted repeat sequence (IR) driven by UAS (Upstream Activation Sequence) were prepared in the National Institute of Genetics (NIG) in Japan, as previously described.^36^ Briefly, a cDNA fragment with nucleotide position 1–500 of the coding sequence was obtained by PCR and was inserted as an IR in a head-to-head manner into a modified Bluescript vector, pSC1. Then IR-fragments were excised by *Not*I and were subcloned into pUAST, a germline transformation vector containing UAS.^37^

In the earlier stages of this research, we did not select the IR strains but randomly employed them according to the order in which NIG collected them. In the later stages, we preferentially focused on 302 genes that were predicted to encode secretory proteins.^38^ Each *UAS-IR* fly strain was crossed with another strain carrying *ap-GAL4*, *UAS-GFP* and *puc-lacZ.* The offspring larvae possessing these four transgenes were reared at 25 °C on a standard diet and then dissected at the late third instar larval stage for immunological staining.

### Immunological staining

The dissected carcasses with the imaginal discs were fixed in 4% formaldehyde for 20 min at room temperature and washed with PBS (phosphate buffered saline) containing Triton X-100 (0.02%). The *puc-lacZ* expression was detected by indirect immunofluorescence using the murine anti-β-galactosidase antibody (Promega, #Z378B, 1/200 dilution). Active Caspase-3 was detected by the rabbit anti-cleaved Caspase-3 antibody (Cell Signaling Technology, #9661, 1/200 dilution), which is known to bind to the cleaved (activated) forms of mammalian Caspase-3 and its *Drosophila* homolog Drice. DIAP1 was detected by the rabbit anti-DIAP1 antibody (1/500 dilution).^39^ Specific binding of these primary antibodies was visualized by fluorescent secondary antibodies, such as the anti-mouse Ig-Cy3 (#715–165–151, Jackson Immunoresearch) and anti-rabbit Ig-Cy5 (#711–175–152, Jackson Immunoresearch) antibodies. Incubation with these primary or secondary antibodies was performed at 37 °C for 1 hour or at 4 °C overnight. Microscopic observation was performed without antifade reagents by the Leica deconvolution system Q550FW.

### Prediction of secreted proteins in the *Drosophila* genome

Putative secreted proteins were searched based on the presence of hydrophobic residues in the N-terminal amino acids. When the average hydrophobicity index in the 25 amino acids between positions 6 and 30 exceeded 0.953, the protein was assumed to be secreted. The predicted protein data set from BDGP release 4.2 was searched using the program “Ahiru”,^38^ which was written based on the algorithm described in^40^ (http://bioinformatics.oxfordjournals.org/cgi/reprint/18/2/298); this yielded a list of 2,184 candidate genes encoding secreted proteins. Among them, 296 genes available in the RNAi strains in NIG were used for the screen.

### How to access the immunofluorescence images

To access the fluorescent images, go to the middle of the right column of the web page (http://www.shigen.nig.ac.jp/fly/nigfly/index.jsp), and click the line of “Browse All RNAi Stocks”. In the newly appeared page, you can see all of the IR strains. When you click each Stock ID name which has the “wing disc” icon on the right column, you can see a set of immunofluorescence images at the bottom of the further next page.
